# Self-Assembly and Drug Release Capacities of Organogels via Some Amide Compounds with Aromatic Substituent Headgroups

**DOI:** 10.3390/ma9070541

**Published:** 2016-07-04

**Authors:** Lexin Zhang, Tifeng Jiao, Kai Ma, Ruirui Xing, Yamei Liu, Yong Xiao, Jingxin Zhou, Qingrui Zhang, Qiuming Peng

**Affiliations:** 1 Hebei Key Laboratory of Applied Chemistry, School of Environmental and Chemical Engineering, Yanshan University, Qinhuangdao 066004, China; zhanglexin@ysu.edu.cn (L.Z.); makai@ipe.ac.cn (K.M.); rrxing@ipe.ac.cn (R.X.); liuym@ipe.ac.cn (Y.L.); zhoujingxin@ysu.edu.cn (J.Z.); zhangqr@ysu.edu.cn (Q.Z.); 2 State Key Laboratory of Metastable Materials Science and Technology, Yanshan University, Qinhuangdao 066004, China; pengqiuming@ysu.edu.cn; 3 Environmental Protection Sciences Research Institute of Qinhuangdao City, Qinhuangdao 066001, China; 13603357776@163.com

**Keywords:** nanostructure, self-assembly, organogel, amide derivative, substituent headgroup, drug release

## Abstract

In this work, some amide compounds with different aromatic substituent headgroups were synthesized and their gelation self-assembly behaviors in 22 solvents were characterized as new gelators. The obtained results indicated that the size of aromatic substituent headgroups in molecular skeletons in gelators showed crucial effect in the gel formation and self-assembly behavior of all compounds in the solvents used. Larger aromatic headgroups in molecular structures in the synthesized gelator molecules are helpful to form various gel nanostructures. Morphological investigations showed that the gelator molecules can self-assembly and stack into various organized aggregates with solvent change, such as wrinkle, belt, rod, and lamella-like structures. Spectral characterizations suggested that there existed various weak interactions including π-π stacking, hydrogen bonding, and hydrophobic forces due to aromatic substituent headgroups and alkyl substituent chains in molecular structures. In addition, the drug release capacities experiments demonstrated that the drug release rate in present obtained gels can be tuned by adjusting the concentrations of dye. The present work would open up enormous insight to design and investigate new kind of soft materials with designed molecular structures and tunable drug release performance.

## 1. Introduction

The self-assembly technique is a powerful approach for functionalized small molecules into supramolecular nanostructures and important for the development of new nanoscale materials and composites [1,2,3,4,5,6]. As a kind of soft material, organized supramolecular gels from low weight molecular organic gelators or polymers could show various nanostructures through weak interactions including hydrophobic interaction, π-π interactions, hydrogen bonds, and van der Waals forces [7,8,9,10]. For examples, in recent years Huang’s group has achieved excellent research works on the investigation of supramolecular gels constructed from macrocycle-based host-guest molecular recognition motifs [11,12,13,14,15,16]. Therein various methods for characterizing supramolecular gels, structure control, stimuli-responsiveness, and applications in various areas are discussed, and future research directions are proposed. In addition, supramolecular gels have got more attention in soft matter areas due to the important scientific contributions and potential wide applications, such as controlled drug release, medical implants, nanomaterials templates, and biosensors [17,18,19,20,21,22]. The formed 3D networks within the supramolecular gels can also demonstrate the promising possibility to construct biosensors, smart actuators, and other molecular switches [23,24,25,26].

In addition, controlled drug release behaviors from self-assembly nanostructures for drug therapy have attracted more attention, which can demonstrate new properties, such as a wide therapeutic window, low toxicity, and good drug release efficacy [27,28,29,30,31]. In particular, controlled drug release from hydrogel systems could release various drugs according to external stimuli factors, such as ionic strength, temperature, pH, magnetic field, etc. [32,33,34,35,36]. Among various gels, the modification of functional substituted groups into molecular structures could produce new structures and new features are expected. In our previous reports, the gelation behaviors of some cholesterol amide compounds including photo-responsive azobenzene substituent groups and cholesteryl segments have been characterized [37,38]. In the following work, the gelation behaviors of designed bolaform cholesteryl derivatives with different spacers and amide compounds with luminol/azobenzene substituent segments have been characterized [39,40,41,42]. Therein we have characterized the effects of substituent spacer and chain on the nanostructures of obtained organogels and investigated various kinds of weak interactions in gel formation process. Moreover, in other recent reports, we have demonstrated the preparation of GO composite hydrogels as good dye adsorbents/catalysts and injectable collagen-based hydrogels for drug delivery and sustained release [20,43,44].

**Figure 1:**
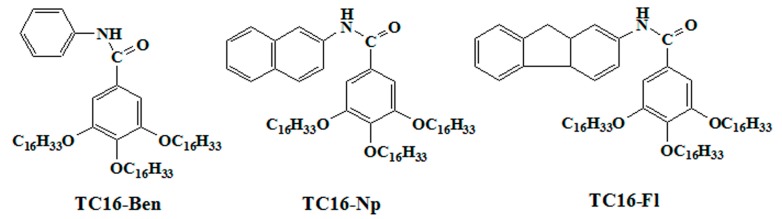
Molecular structures and abbreviations of three obtained amide derivatives.

In the present work, we have prepared the supramolecular gels from new amide compounds with different aromatic substituent headgroups. The molecular structures and abbreviations of obtained amide derivatives were shown in Figure 1. In molecular skeletons, the headgroups with different sizes were attached to a benzene ring via amide bond to form a rigid and hydrophobic substituent segment. The obtained results indicated that all compounds could fabricate different organogels in presently used solvents. Morphological characterization of the organogels showed various nanostructures of the aggregates in the gels. In addition, the drug release behaviors with different dye concentrations were also investigated.

## 2. Results and Discussion

### 2.1. Preparation and Characterization of Supramolecular Gels

**Figure 2:**
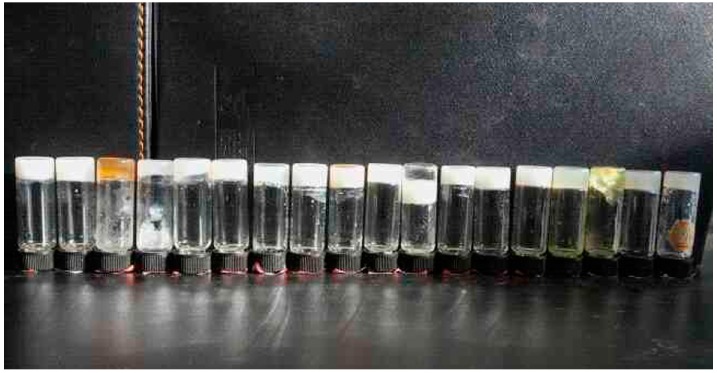
Photograph of TC16-Fl organogels in present used solvents (from left to right, nitrobenzene, acetone, DMF, aniline, pyridine, petroleum ether, n-hexane, ethanol, n-propanol, isopropanol, isooctanol, n-butanol, n-butyl acrylate, cyclohexanone, n-pentanol, 1,4-dioxane, cyclopentanone, and isopentanol).

The gelation behaviors and the critical gelation concentrations of three obtained amide compounds in 22 solvents are shown in Table 1. The results in the table indicated that all compounds were efficient gelators for some present solvents. Firstly, 3,4,5-tris(alkyloxy)benzoic amide compound with phenyl substituent headgroup (abbreviated as TC16-Ben) in molecular structure can form gels in nine solvents, such as aniline, petroleum ether, n-hexane, ethanol, n-propanol, isopropanol, n-butanol, n-pentanol, and isopentanol. As for 3,4,5-tris(alkyloxy)benzoic amide compound with naphthyl substituent headgroup (abbreviated as TC16-Np) in molecular skeleton, seven kinds of used solvents can be observed to form gels, including dimethylformamide (DMF), aniline, n-propanol, n-butanol, n-pentanol, 1,4-dioxane, and isopentanol. In addition, for the case of 3,4,5-tris(alkyloxy)benzoic amide compound with larger fluorenyl substituent headgroup (abbreviated as TC16-Fl) in molecular structure, the number of obtained gels changed to 18. In addition, the temperatures of gel to sol transitions (*T*_g_) ranged from 60 to 65 °C. The photograph of obtained gels from TC16-Fl was demonstrated in Figure 2. The above data suggested that aromatic substituent headgroups demonstrated an important effect upon the gelation behaviors of present designed compounds. It indicated that larger aromatic headgroup in molecular structures in functional gelators are helpful to self-assembly in organized manners and subsequently produce gelation of organic solvents, which was in good accordance with previous relative reports [45,46]. Moreover, it is worthy to note that present three compounds can form organogels in aniline, n-propanol, n-butanol, n-pentanol, and isopentanol, respectively, which can originate from the weak intermolecular forces between amide compounds and solvents. The reasons for the strengthening on the gelation phenomena for TC16-Fl can be due to the change of π-π stacking and spatial conformation of the gelators with a large fluorenyl substituent headgroup in the skeleton.

**Figure 3:**
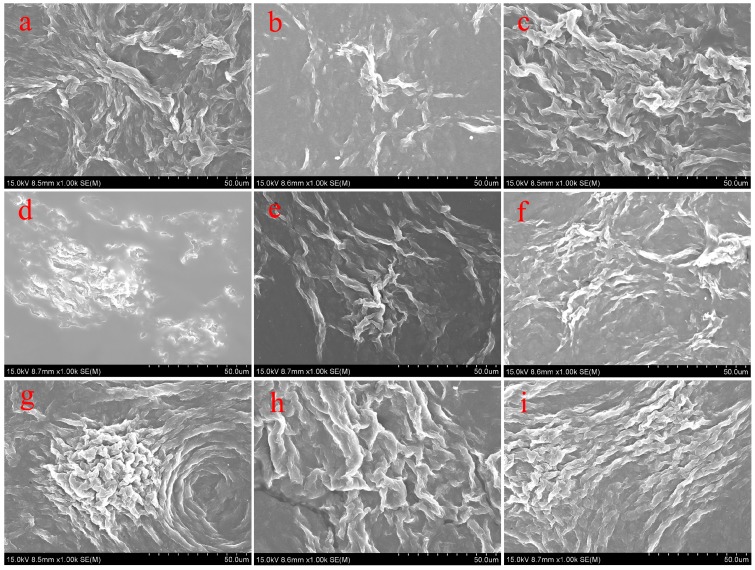
SEM images of TC16-Ben xerogels from gels in various solvents: (**a**) aniline; (**b**) petroleum ether; (**c**) n-hexane; (**d**) ethanol; (**e**) n-propanol; (**f**) isopropanol; (**g**) n-butanol; (**h**) n-pentanol; (**i**) isopentanol.

**Figure 4:**
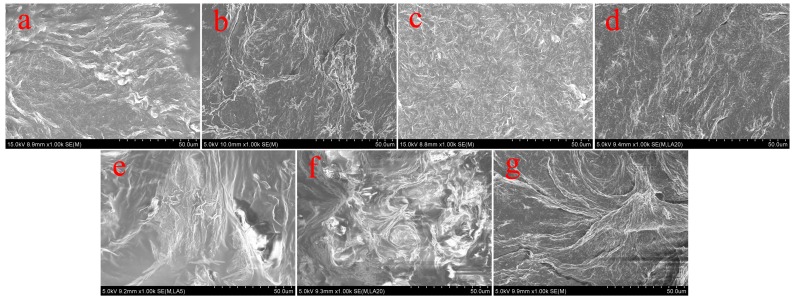
SEM images of TC16-Np xerogels from gels in various solvents: (**a**) DMF; (**b**) aniline; (**c**) n-propanol; (**d**) n-butanol; (**e**) n-pentanol; (**f**) 1,4-dioxane; (**g**) isopentanol.

**Figure 5:**
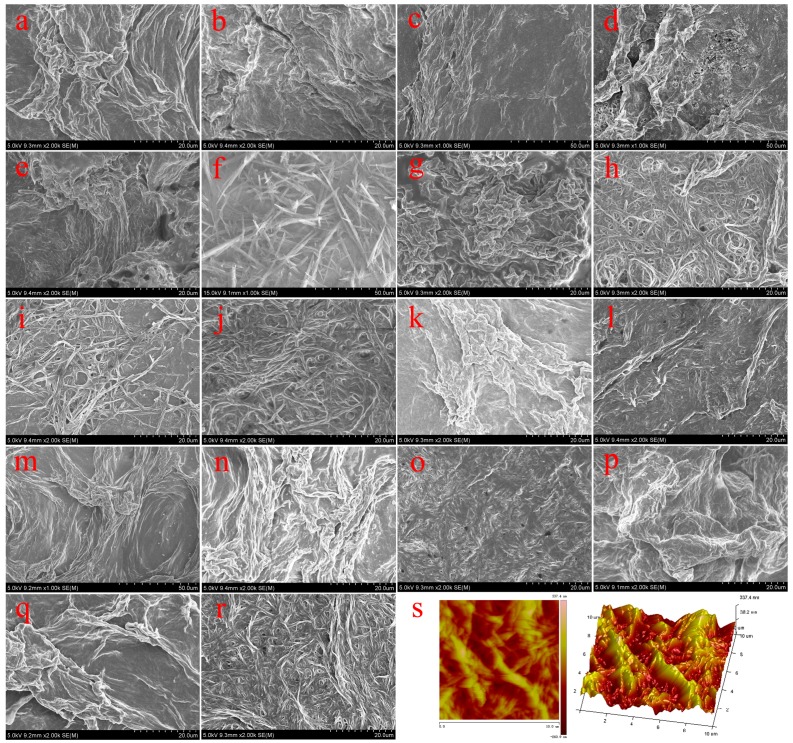
SEM images of TC16-Fl xerogels from gels in various solvents: (**a**) nitrobenzene; (**b**) acetone; (**c**) DMF; (**d**) aniline; (**e**) pyridine; (**f**) petroleum ether; (**g**) n-hexane; (**h**) ethanol; (**i**) n-propanol; (**j**) isopropanol; (**k**) isooctanol; (**l**) n-butanol; (**m**) n-butyl acrylate; (**n**) cyclohexanone; (**o**) n-pentanol; (**p**) 1,4-dioxane; (**q**) cyclopentanone; (**r**) isopentanol. Pictures in (**s**) indicate AFM images in 2D height and 3D model of TC16-Fl xerogel from gel in n-pentanol.

In order to characterize the obtained nanostructures in organogels, the morphologies of the xerogels were investigated by SEM and AFM techniques. From images shown in Figure 3, it was clearly demonstrated that the TC16-Ben xerogels mainly showed large wrinkle-like or lamella-like aggregates with micrometer scale. Moreover, as shown in Figure 4, the SEM images of xerogels from TC16-Np gels displayed more nanostructures, such as fiber, wrinkle, and lamella. Figure 5 showed the SEM images of TC16-Fl xerogels from 18 solvents, which demonstrated the self-assembled diverse micro/nano- morphologies included various nano-aggregates. The formed aggregates showed the tendency to overlap each other and stack to fabricate larger multi-level aggregations. Moreover, as shown in Figure 5s, from the AFM images in 2D height and 3D model of TC16-Fl xerogel from gel in n-pentanol, it is clearly observed that these organized fiber aggregates included smaller domains/particles by self-assembly of the present amide compounds. The shown morphologies of the aggregates may be explained in that common weak and directional intermolecular interactions, such as π-π stacking or hydrophobic interactions, are helpful to form belt or fiber-like nanostructures [47,48]. The morphological changes in gels can be mainly attributed to the various strengths of the intermolecular π-π stacking between aromatic segments, demonstrating important role in changing organized self-assembly models and formation of new aggregates.

**Figure 6:**
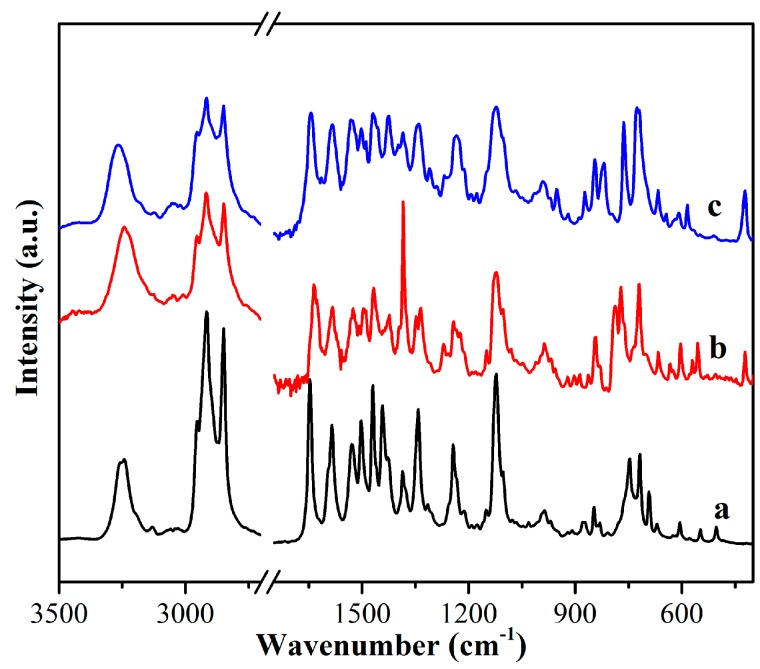
FT-IR spectra of xerogels from gels in n-pentanol: (**a**) TC16-Ben; (**b**) TC16-Np; (**c**) TC16-Fl.

In addition, it is well-known that Fourier transform infrared (FT-IR) spectra show an important role in the characterizations of organogels [49,50,51]. In the present case, to further illustrate the effect of substituent headgroups on self-assembly models, the IR spectra of xerogels of TC16-Ben, TC16-Np, and TC16-Fl from n-pentanol were characterized and compared, as shown in Figure 6. As for the spectrum of TC16-Ben xerogel, some characteristic peaks appeared at 3246, 2918, 2848, 1646, 1585, and 1469 cm^−1^, which could be assigned to the N–H stretching, methylene stretching, amide I band, and methylene shearing, respectively [52,53,54]. In addition, the IR spectra of TC16-Np and TC16-Fl xerogels changed obviously. One is the position of N–H stretching shifted to 3237 and 3263 cm^−1^, respectively. Another change was the band attributed to amide I band shifted to 1634 and 1643 cm^−1^, respectively. The obtained spectral results indicated the existence of functional groups and the formation of different H-bonds between amide groups in obtained gels.

**Figure 7:**
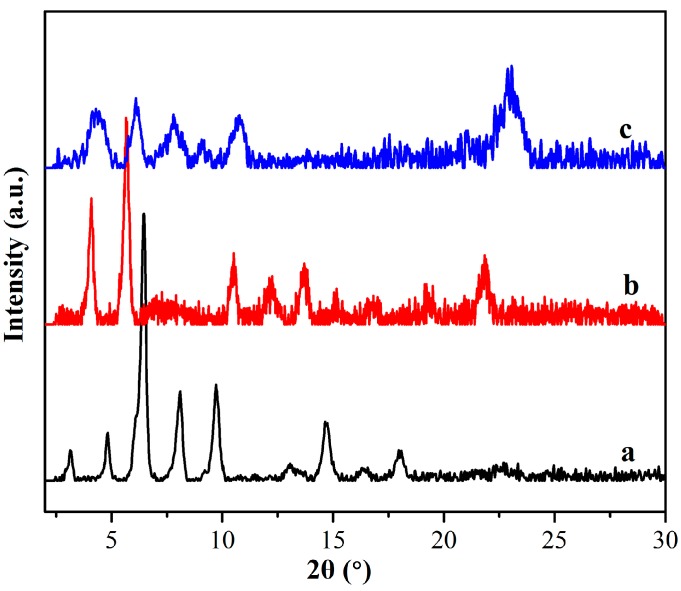
X-ray diffraction patterns of xerogels from gels in n-pentanol: (**a**) TC16-Ben; (**b**) TC16-Np; (**c**) TC16-Fl.

**Figure 8:**
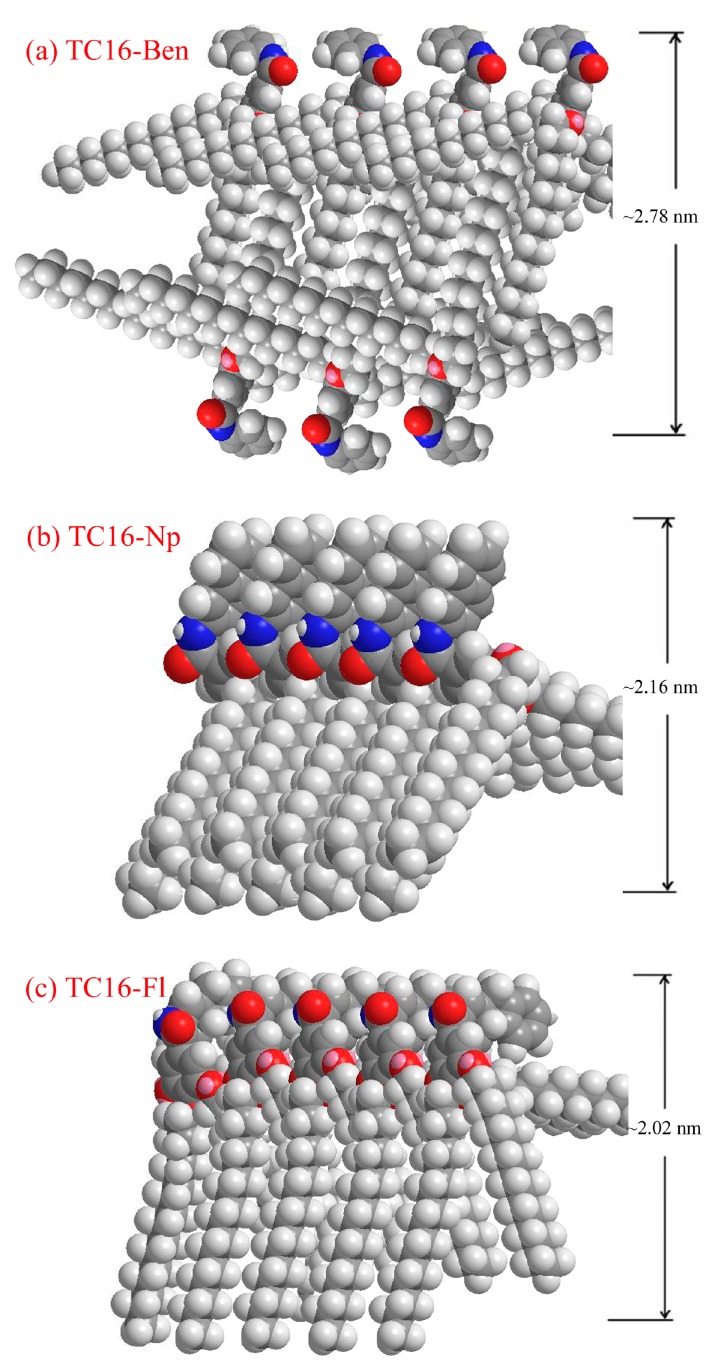
Schematic pictures of rational assembly modes for present amide compounds in gels: (**a**) TC16-Ben; (**b**) TC16-Np; (**c**) TC16-Fl.

In addition, in order to further investigate the self-assembly nanostructures in xerogels, the XRD patterns of all xerogels from gels in n-pentanol were characterized. Firstly, as shown in Figure 7, the typical curve for TC16-Ben sample from n-pentanol shows main peaks in the angle region (2θ values, 3.18°, 4.81°, 6.45°, 8.12°, 9.75°, and 14.70°) corresponding to *d* values of 2.78, 1.84, 1.37, 1.09, 0.91, and 0.60 nm, respectively. In addition, the XRD data of TC16-Np and TC16-Fl xerogels from n-pentanol were compared, indicating obvious changes. The minimum diffraction peaks at 4.10° and 4.37° demonstrated the corresponding *d* values of 2.16 and 2.02 nm, respectively. The shift of corresponding *d* values demonstrated various assembly units with different nanostructures in the obtained gels [55,56]. Considering the XRD results described above and the organized packing in these organogels, some possible packing modes of these gelators were proposed and schematically shown in Figure 8. As for TC16-Ben gels, due to the flexibility and hydrophobic forces of long alkyl chains in the molecular skeleton, after the intermolecular orderly stacking in different solvents, the interdigitated structures were obtained. So the corresponding *d* values of 2.78 nm were obtained from different solvents. This phenomenon is similar to our previous results for interfacial self-assembly of gemini-type amphiphiles with a hydrophobic spacer at the air/water interface [57]. As for TC16-Np and TC16-Fl with naphthyl or fluorenyl substituent headgroup in molecular structure, due to the strong π-π stacking, the combination of a flexible ether band and a rigid aromatic segment in the molecular spacer with π-π stacking seemed more suitable to adjust molecular conformation to self-assemble and form organized stacking nanostructures. The obtained experimental values of TC16-Np and TC16-Fl in gels were 2.16 and 2.02 nm, which was near the calculated molecular length, suggesting cooperative stacking in an organized way to form various nanostructures. Meanwhile, it should be noted that this phenomenon can be compared with the results of our recent works [40,41,42]. Therein, functionalized amide derivatives with the substituent groups of cholesteryl, azobenzene, and luminol residues can have a profound effect on the gelation abilities and the as-formed nanostructures of the studied compounds. The present results described indicated again that the aromatic substituent headgroups had showed great effect on the self-assembly process in gel formation.

### 2.2. Drug Release Properties of Supramolecular Gels

**Figure 9:**
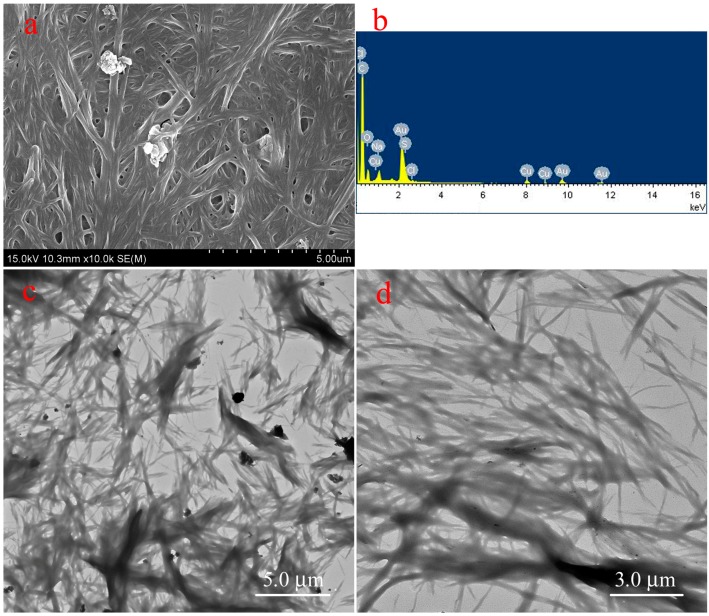
Scanning electron microscope (SEM) (**a**) and transmission electron microscope (TEM) (**c**) images of TC16-Fl xerogels from gels in n-pentanol with the addition of CR (2 mg/mL) in comparison with TEM image (**d**) of same gel without CR. Image (**b**) indicate energy dispersive X-ray spectroscopy (EDXS) taken on the nanoparticle shown in picture (**a**).

**Figure 10:**
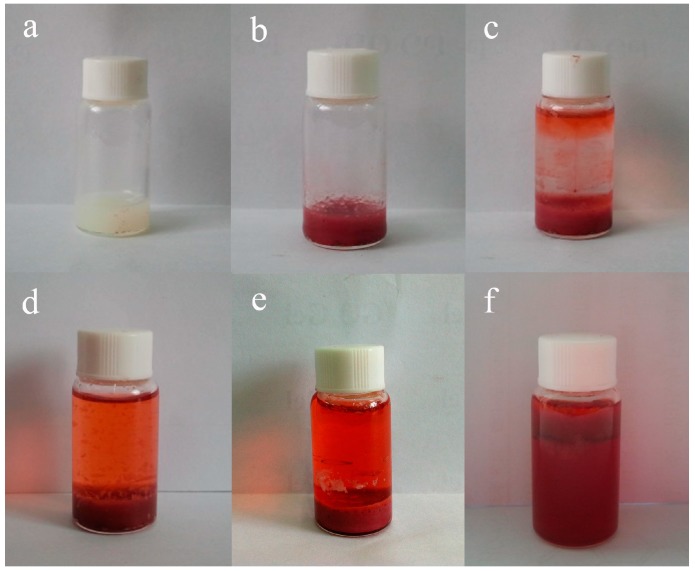
Photographs of release process of TC16-Fl organogels in n-pentanol with addition of CR (2 mg/mL): (**a**) as-formed organogel; (**b**) gel with addition of CR; (**c**–**f**) with water time of 10, 50, 200, and 500 min, respectively.

**Figure 11:**
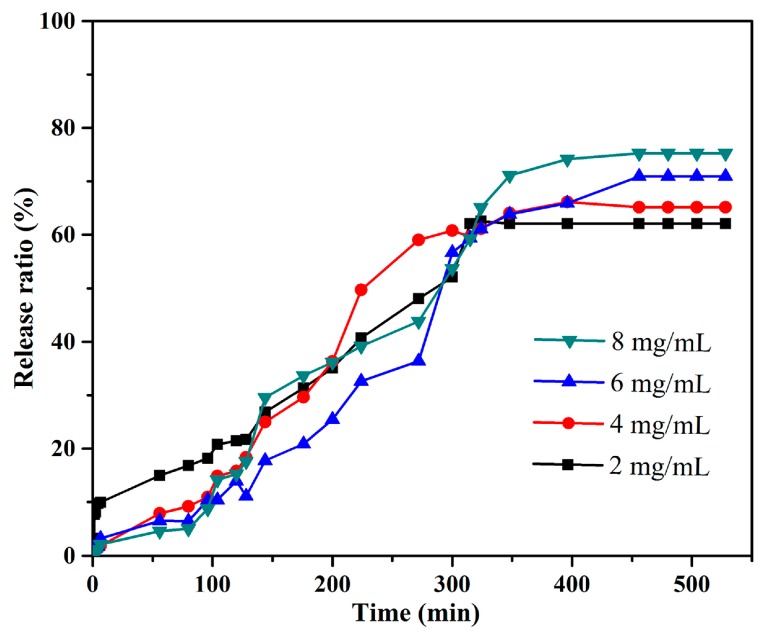
Release capacities of TC16-Fl organogels in n-pentanol with different CR concentrations.

**Figure 12:**
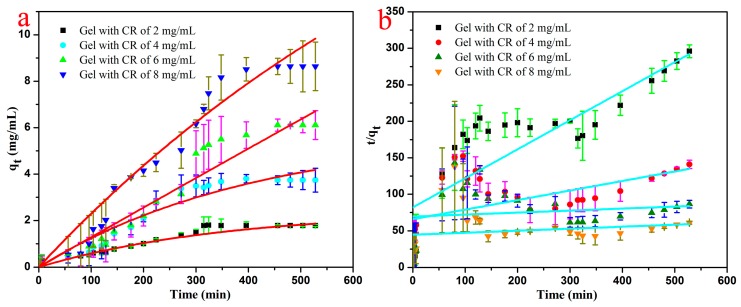
Release kinetics curves of as-prepared TC16-Fl organogels in n-pentanol with different CR concentrations at 298 K: (**a**) pseudo-first-order model; (**b**) pseudo-second-order model.

Because the organized gels can load various drugs due to 3D porous structures, it is convenient to characterize their drug release capacities. Considering their unique nanostructures and solvents, the typical TC16-Fl gels in n-pentanol with addition of Congo red (CR) as model drug were preferred and their drug release properties upon time were investigated. As can be clearly observed in Figure 9, some drug particles appeared within the fiber nanostructure of gels, which were confirmed by the EDXS data with S element in CR molecule. In addition, the TEM images of same gel with and without CR also clearly indicated the same results. It is worthy to note that the addition of release time is crucial and applicable in actual drug therapy due to the necessity of drug efficacy [58]. Next, the release process of TC16-Fl gel in n-pentanol with CR concentration of 2 mg/mL was characterized, with photographs at different time intervals shown in Figure 10. It can be clearly observed that with time, the color of the upper water solution changed to deeper, suggesting the release of CR molecules from gel to water phase. In addition, the release capacities of TC16-Fl gels in n-pentanol with different CR concentrations were also demonstrated, as shown in Figure 11. It was found that the release rates before 320 min seemed random increment with change of CR concentration, which could be mainly attributed to the original diffusion process of composited CR molecules from the outermost layer of TC16-Fl gels. However, the final release ratios were 75.2%, 70.9%, 65.1%, and 62.2% for the CR concentrations of 8, 6, 4, and 2 mg/mL, respectively. Moreover, Figure 12 demonstrated the release kinetics curves of as-prepared TC16-Fl organogels in n-pentanol with different CR concentrations at 298 K. Classical kinetic models were employed to describe the above release process as the following formulas:

The pseudo-first-order model:

(1) $$\log(q_{e}-q_{t})=(\log q_{e})-\frac{k}{2.303}t$$

The pseudo-second-order model:

(2) $$\frac{t}{q_{t}}=\frac{1}{k{q_{e}}^{2}}+\frac{t}{q_{e}}$$

where *q_e_* and *q_t_* represent the amount of dye released (mg/mL) at equilibrium and time t, respectively, and the *k* values are the kinetic rate constants. The present kinetic data for dyes release (Table 2) indicated that the pseudo-first-order model could better described the present release process with a high correlation coefficient (all of *R*^2^ values >0.94). Such kinetic behaviors can also be associated with the unique gel nanostructures. Therefore, the drug release capacities of the obtained TC16-Fl gels can also be adjusted by altering the release times and drug concentrations.

## 3. Experimental Section

### 3.1. Materials and Reagents

The starting materials, aniline, β-naphthylamine, 2-aminofluorene, methyl 3,4,5-trihydroxybenzoate, 1-bromohexadecane, and Congo red (CR) were purchased from Aladdin Chemicals, Alfa Aesar (Tianjin, China) Chemicals, and TCI Chemicals (Shanghai, China), respectively. Other used reagents were all for analysis purity from Aladdin Chemicals and used as received. The solvents were purchased from Beijing Chemicals (Beijing, China) and were distilled before use. Deionized water was used in all steps. 3,4,5-tris(alkyloxy)benzoic acid with alkyl substituent chains were synthesized according to previous report [59] and confirmed by ^1^H-NMR. Then three amide compounds with aromatic substituent headgroups were synthesized by similar methods [40,41]. Simply speaking, first of all, 3,4,5-tris(alkyloxy)benzoic acid chloride was synthesized by heating benzoic acid-containing compound solution in sulfoxide chloride and dichloromethane (*v*/*v* = 1:1) for 10 h at 70 °C. Then the prepared benzoic acid chloride reacted with three different aromatic amines in dried dichloromethane at the presence of pyridine for 3–4 days at room temperature. After that step, the obtained mixtures were washed with dilute hydrochloric acid and pure water with several times, filtered, and dried in vacuum. The solid residues were purified by recrystallization in ethanol solution as pale or yellow solids. The final products and their abbreviations are shown in Figure 1, which were confirmed by ^1^H-NMR and elemental analysis. TC16-Ben: 73% yield; ^1^H-NMR (400 MHz, CDCl_3_, 25 °C) δ (ppm) 7.60–7.38 (ArH, 4H), 6.89–6.82 (ArH, 3H), 6.46 (s, NH, 1H), 4.02–3.98 (t, -OCH_2_CH_2_-, 6H), 1.84–1.72 (m, -OCH_2_CH_2_-, 6H), 1.45–1.26 (m, -CH_2_-, 78H), 0.89–0.85 (t, -CH_3_, 9H). Anal. Calcd. for C_61_H_107_NO_4_: C, 79.77; H, 11.74; N, 1.52. Found: C, 79.85; H, 11.68; N, 1.59. TC16-Np: 81% yield; ^1^H-NMR (400 MHz, CDCl_3_, 25 °C) δ (ppm) 8.60–8.52 (ArH, 2H), 7.85–7.74 (ArH, 2H), 7.55–7.44 (ArH, 2H), 6.59–6.53 (ArH, 3H), 6.51 (s, NH, 1H), 4.05–4.00 (t, -OCH_2_CH_2_-, 6H), 1.83–1.73 (m, -OCH_2_CH_2_-, 6H), 1.47–1.22 (m, -CH_2_-, 78H), 0.89–0.85 (t, -CH_3_, 9H). Anal. Calcd. for C_65_H_109_NO_4_: C, 80.60; H, 11.34; N, 1.45. Found: C, 80.85; H, 11.22; N, 1.49. TC16-Fl: 80% yield; ^1^H-NMR (400 MHz, CDCl_3_, 25 °C) δ (ppm) 7.85–7.73 (ArH, 2H), 7.55–7.33 (ArH, 2H), 7.25–7.14 (ArH, 2H), 6.69–6.58 (ArH, 3H), 6.50 (s, NH, 1H), 4.04–3.94 (t, -OCH_2_CH_2_-, -CH_2_-, 8H), 1.83–1.78 (m, -OCH_2_CH_2_-, 6H), 1.49–1.26 (m, -CH_2_-, 78H), 0.91–0.86 (t, -CH_3_, 9H). Anal. Calcd. for C_68_H_113_NO_4_: C, 80.97; H, 11.29; N, 1.39. Found: C, 81.05; H, 11.23; N, 1.35.

### 3.2. Gelation Preparation and Drug Delivery

The present amide compounds were tested to prepare possible gels according to a simple procedure. Firstly, a weighted amount of gelator molecules and a measured volume of selected organic solvent were placed into a sealed glass bottle and the solution was and the solution ultrasonicated in a sonic bath for 15 min in order to obtain good dispersion. After that, the solution heated in a water bath at temperatures of 80 °C for 15 min. Then, the solution was cooled to room temperature in air and the test bottle was inverted to see if a gel was formed. When the gelator formed a gel by immobilizing the solvent at this stage, it was denoted as “G”. For the systems in which only solution remained until the end of the tests, they were referred to as solution (S). The system in which the potential gelator could not be dissolved even at the boiling point of the solvent was designated as an insoluble system (I). Critical gelation concentration refers to the minimum concentration of the gelator for gel formation.

For drug release experiment, the process was processing at room temperature. The CR dye was chosen to use as a model drug molecule. First of all, in order to load CR molecules, different CR concentrations (2, 4, 6, and 8 mg/mL) were obtained in TC16-Fl organogels in n-pentanol. Then, for the drug release experiments, the release behaviors of CR were determined with 752 UV-vis spectrophotometer (Sunny Hengping scientific instrument Co., Ltd., Shanghai, China) at the wavelength of 497 nm at a function of time with the pre-established calibration curves, respectively. The typical experimental procedures were demonstrated as follows: the above CR-loaded gels were kept immersed in 5 mL deionized water at room temperature. At different time intervals, the supernatant solution was collected for measurement by UV spectrophotometer. Each experiment point was carried out in triplicate. Release ratio % = (the amount of CR released from gels)/(the total amount of CR loaded by gels) × 100%.

### 3.3. Measurements

Firstly, these as-formed xerogels were obtained by freeze drying at low temperature (−50 °C) after 2–3 days. The obtained dried samples were attached to different substrates, such as mica, copper foil, glass, and CaF_2_ slice for morphological and spectral investigation, respectively. Before SEM measurement, the samples were coated on copper foil fixed by conductive adhesive tape and shielded by gold. The morphologies of all lyophilized samples were characterized by using both a field-emission scanning electron microscopy (FE-SEM, S-4800II, Hitachi, Japan) with the accelerating voltage of 5–15 kV and a transmission electron microscopy (TEM, HT7700, Hitachi High-Technologies Corporation, Edo, Tokyo, Japan). The chemical composition of the samples was characterized by energy-dispersive X-ray spectroscopy (EDXS). EDXS analysis was typically performed at an accelerating voltage of 200 kV, using an Oxford Link-ISIS X-ray EDXS microanalysis system attached to SEM. AFM images were recorded using a Nanoscope VIII Multimode Scanning Probe Microscope (Veeco Instrument, Plainview, NY, USA) with silicon cantilever probes. All AFM images were shown in the height mode without any image processing except flattening. Transmission FT-IR spectra of the xerogel were obtained by Nicolet is/10 FT-IR spectrophotometer from Thermo Fisher Scientific Inc. by average 32 scans and at a resolution of 4 cm^−1^. The XRD measurement was conducted using a Rigaku D/max 2550PC diffractometer (Rigaku Inc., Tokyo, Japan). The XRD pattern was obtained using CuKα radiation with an incident wavelength of 0.1542 nm under a voltage of 40 kV and a current of 200 mA. The scan rate was 0.5°/min. The elemental analysis was carried out with the Flash EA Carlo-Erba-1106 Thermo-Quest. ^1^H-NMR spectra were obtained on an ARX400 (Bruker, Fällanden, Switzerland) NMR spectrometer in CDCl_3_ with TMS as an internal standard.

## 4. Conclusions

In summary, we successfully demonstrated the design and preparation of organogels via three amide derivatives with aromatic substituent headgroups. Their gelation behaviors in used organic solvents can be altered by regulating the size of aromatic headgroups and used solvents. The experimental results indicated that the larger substituent headgroups in obtained amide compounds were favorable for gel formation in used solvents. Morphological characterization suggested that the gelator molecules could fabricate by self-assembly into different aggregates with belt, rod, lamella, and wrinkle-like nanostructures with solvent changes. Spectral investigation demonstrated that various weak interactions existed, such as H-bond and π-π stacking, depending on the substituent headgroups in molecular structures. Experimental results of drug release capacities indicated that the drug release rate of the TC16-Fl gel can be regulated by the release times and drug concentrations. It is believed that present research work will attract more attention for gel research and applications due to the adjustable preparation strategy and drug delivery capacities.

## Figures and Tables

**Table 1:** Gelation behaviors of three amide derivatives at room temperature. ^1^

Solvents	TC16-Ben	TC16-Np	TC16-Fl
Nitrobenzene	S	S	G (2.0)
Acetone	PS	S	G (2.0)
DMF	PS	G (2.5)	G (2.5)
Aniline	G (1.5)	G (2.5)	G (2.0)
Pyridine	PS	S	G (2.5)
Petroleum ether	G (1.5)	PS	G (2.0)
Ethanolamine	I	PS	I
n-Hexane	G (1.5)	PS	G (2.0)
Ethyl acetate	PS	PS	PS
Ethanol	G (2.0)	S	G (2.0)
DMSO	S	S	S
n-Propanol	G (1.5)	G (2.0)	G (2.5)
Isopropanol	G (1.5)	PS	G (2.5)
Isooctanol	S	PS	G (2.0)
n-Butanol	G (1.5)	G (2.0)	G (2.0)
n-Butyl acrylate	I	PS	G (2.5)
Cyclohexanone	S	S	G (2.5)
n-Pentanol	G (1.5)	G (2.0)	G (2.0)
1,4-Dioxane	PS	G (2.0)	G (2.0)
Cyclopentanone	PS	PS	G (2.0)
THF	PS	PS	PS
Isopentanol	G (2.0)	G (2.5)	G (2.5)

^1^ DMF, dimethylformamide; DMSO, dimethyl sulfoxide; THF, tetrahydrofuran; S: solution; PS: partially soluble; G: gel; I: insoluble. For gels, the critical gelation concentrations at room temperature are shown in parentheses, *w*/*v* %.

**Table 2:** Kinetic parameters of the as-prepared TC16-Fl organogels in n-pentanol with different CR concentrations at 298 K (experimental data from Figure 12).

TC16-Fl Organogels in n-Pentanol	Pseudo-First-Order Model			Pseudo-Second-Order Model		
	*q_e_* (mg/mL)	*R*^2^	*k*_1_ (min^−1^)	*q_e_* (mg/mL)	*R*^2^	*k*_2_ (mL/mg·min)
CR concern. (2 mg/mL)	1.8468	0.94511	2.76 × 10^−3^	2.24	0.7117	2.86 × 10^−3^
CR concern. (4 mg/mL)	4.1351	0.94472	2.30 × 10^−3^	7.68	0.24702	2.55 × 10^−4^
CR concern. (6 mg/mL)	6.6846	0.94878	1.84 × 10^−4^	16.83	0.03573	5.86 × 10^−5^
CR concern. (8 mg/mL)	9.8288	0.96162	9.21 × 10^−4^	41.19	0.01986	1.27 × 10^−5^

## References

[1] Delbecq F., Tsujimoto K., Ogue Y., Endo H., Kawai T. (2013). N-stearoyl amino acid derivatives: Potent biomimetic hydro/organogelators as templates for preparation of gold nanoparticles. J. Colloid Interface Sci..

[2] Oh H., Jung B.M., Lee H.P., Chang J.Y. (2010). Dispersion of single walled carbon nanotubes in organogels by incorporation into organogel fibers. J. Colloid Interface Sci..

[3] Wang W., Jiao T., Zhang Q., Luo X., Hu J., Chen Y., Peng Q., Yan X., Li B. (2015). Hydrothermal synthesis of hierarchical core–shell manganese oxide nanocomposites as efficient dye adsorbents for wastewater treatment. RSC Adv..

[4] Basrur V.R., Guo J., Wang C., Raghavan S.R. (2013). Synergistic gelation of silica nanoparticles and a sorbitol-based molecular gelator to yield highly-conductive free-standing gel electrolytes. ACS Appl. Mater. Interfaces.

[5] Xing R., Jiao T., Yan L., Ma G., Liu L., Dai L., Li J., Möhwald H., Yan X. (2015). A colloidal gold-collagen protein core-shell nanoconjugate: One-step biomimetic synthesis, layer-by-layer assembled film and controlled cell growth. ACS Appl. Mater. Interfaces.

[6] Yan N., Xu Z., Diehn K.K., Raghavan S.R., Fang Y., Weiss R.G. (2013). Pyrenyl-linker-glucono gelators. correlations of gel properties with gelator structures and characterization of solvent effects. Langmuir.

[7] George S.J., Ajayaghosh A. (2005). Self-assembled nanotapes of oligo(p-phenylene vinylene)s: Sol-gel-controlled optical properties in fluorescent π-electronic gels. Chem. Eur. J..

[8] Ajayaghosh A., Chithra P., Varghese R. (2007). Self-assembly of tripodal squaraines: Cation-assisted expression of molecular chirality and change from spherical to helical morphology. Angew. Chem. Int. Ed..

[9] Zhang X., Chen Z., Wurthner F. (2007). Morphology control of fluorescent nanoaggregates by co-self-assembly of wedge- and dumbbell-shaped amphiphilic perylene bisimides. J. Am. Chem. Soc..

[10] Boerakker M.J., Botterhuis N.E., Bomans P.H.H., Frederik P.M., Meijer E.M., Nolte R.J.M., Sommerdijk N.A.J.M. (2006). Aggregation behavior of giant amphiphiles prepared by cofactor reconstitution. Chem. Eur. J..

[11] Yu G.C., Yan X.Z., Han C.Y., Huang F.H. (2013). Characterization of supramolecular gels. Chem. Soc. Rev..

[12] Ji X.F., Shi B.B., Wang H., Xia D.Y., Jie K.C., Wu Z.L., Huang F.H. (2015). Supramolecular construction of multifluorescent gels: Interfacial assembly of discrete fluorescent gels through multiple hydrogen bonding. Adv. Mater..

[13] Ji X.F., Jie K.C., Zimmerman S.C., Huang F.H. (2015). A double supramolecular crosslinked polymer gel exhibiting macroscale expansion and contraction behavior and multistimuli responsiveness. Polym. Chem. UK.

[14] Dong S.Y., Zheng B., Wang F., Huang F.H. (2014). Supramolecular polymers constructed from macrocycle-based host-guest molecular recognition motifs. Acc. Chem. Res..

[15] Dong S.Y., Yuan J.Y., Huang F.H. (2014). A pillar[5]arene/imidazolium [2]rotaxane: Solvent-and thermo-driven molecular motions and supramolecular gel formation. Chem. Sci..

[16] Dong S.Y., Zheng B., Xu D.H., Yan X.Z., Zhang M.M., Huang F.H. (2012). A crown ether appended super gelator with multiple stimulus responsiveness. Adv. Mater..

[17] Dai H., Chen Q., Qin H., Guan Y., Shen D., Hua Y., Tang Y., Xu J. (2006). A temperature-responsive copolymer hydrogel in controlled drug delivery. Macromolecules.

[18] Kuroiwa K., Shibata T., Takada A., Nemoto N., Kimizuka N. (2004). Heat-set gel-like networks of lipophilic Co(II) triazole complexes in organic media and their thermochromic structural transitions. J. Am. Chem. Soc..

[19] Aldred M.P., Eastwood A.J., Kelly S.M., Vlachos P., Contoret A.E.A., Farrar S.R., Mansoor B., O’Neill M., Tsoi W.C. (2004). Light-emitting fluorene photoreactive liquid crystals for organic electroluminescence. Chem. Mater..

[20] Guo H., Jiao T., Zhang Q., Guo W., Peng Q., Yan X. (2015). Preparation of graphene oxide-based hydrogels as efficient dye adsorbents for wastewater treatment. Nanoscale Res. Lett..

[21] Xin F., Zhang H., Hao B., Sun T., Kong L., Li Y., Hou Y., Li S., Zhang Y., Hao A. (2012). Controllable transformation from sensitive and reversible heat-set organogel to stable gel induced by sodium acetate. Colloid Surf. A Physicochem. Eng. Asp..

[22] Iwanaga K., Sumizawa T., Miyazaki M., Kakemi M. (2010). Characterization of organogel as a novel oral controlled release formulation for lipophilic compounds. Int. J. Pharm..

[23] Lofman M., Koivukorpi J., Noponen V., Salo H., Sievanen E. (2011). Bile acid alkylamide derivatives as low molecular weight organogelators: Systematic gelation studies and qualitative structural analysis of the systems. J. Colloid Interface Sci..

[24] Jiao T., Wang Y., Zhang Q., Yan X., Zhao X., Zhou J., Gao F. (2014). Self-assembly and headgroup effect in nanostructured organogels via cationic amphiphile-graphene oxide composites. PLoS ONE.

[25] Bastiat G., Plourde F., Motulsky A., Furtos A., Dumont Y., Quirion R., Fuhrmann G., Leroux J.C. (2010). Tyrosine-based rivastigmine-loaded organogels in the treatment of Alzheimer’s disease. Biomaterials.

[26] Miyamoto K., Jintoku H., Sawada T., Takafuji M., Sagawa T., Ihara H. (2011). Informative secondary chiroptics in binary molecular organogel systems for donor-acceptor energy transfer. Tetrahedron Lett..

[27] Slowing I.I., Vivero-Escoto J.L., Wu C.W., Lin V.S.Y. (2008). Mesoporous silica nanoparticles as controlled release drug delivery and gene transfection carriers. Adv. Drug Deliv. Rev..

[28] Eeckman F., Moës A.J., Amighi K. (2002). Evaluation of a new controlled-drug delivery concept based on the use of thermoresponsive polymers. Int. J. Pharm..

[29] Xing R., Jiao T., Liu Y., Ma K., Zou Q., Ma G., Yan X. (2016). Co-assembly of graphene oxide and albumin/photosensitizer nanohybrids towards enhanced photodynamic therapy. Polymers.

[30] Kumar C.S.S.R., Mohammad F. (2011). Magnetic nanomaterials for hyperthermia-based therapy and controlled drug delivery. Adv. Drug Deliv. Rev..

[31] Zhang R., Xing R., Jiao T., Ma K., Chen C., Ma G., Yan X. (2016). Carrier-free, chemo-photodynamic dual nanodrugs via self-assembly for synergistic antitumor therapy. ACS Appl. Mater. Interfaces.

[32] Ma K., Jiao T., Shen X., Zhang Q., Li X., Gao F. (2015). Binary organogels via some aminobenzimidazole/benzothiazole compounds and fatty acids with different alkyl lengths: Self-assembly and drug release properties. Integr. Ferroelectr..

[33] Karbarz M., Hyk W., Stojek Z. (2009). Swelling ratio driven changes of probe concentration in pH-and ionic strength-sensitive poly (acrylic acid) hydrogels. Electrochem. Commun..

[34] Ang K.L., Venkatraman S., Ramanujan R.V. (2007). Magnetic PNIPA hydrogels for hyperthermia applications in cancer therapy. Mater. Sci. Eng. C.

[35] Wang L., Liu M., Gao C., Ma L., Cui D. (2010). A pH-, thermo-, and glucose-, triple-responsive hydrogels: Synthesis and controlled drug delivery. React. Funct. Polym..

[36] Zhang Z., Chen L., Zhao C., Bai Y., Deng M., Shan H., Zhuang X., Chen X., Jing X. (2011). Thermo-and pH-responsive HPC-g-AA/AA hydrogels for controlled drug delivery applications. Polymer.

[37] Jiao T.F., Wang Y.J., Gao F.Q., Zhou J.X., Gao F.M. (2012). Photoresponsive organogel and organized nanostructures of cholesterol imide derivatives with azobenzene substituent groups. Prog. Nat. Sci..

[38] Jiao T.F., Gao F.Q., Wang Y.J., Zhou J.X., Gao F.M., Luo X.Z. (2012). Supramolecular gel and nanostructures of bolaform and trigonal cholesteryl derivatives with different aromatic spacers. Curr. Nanosci..

[39] Jiao T.F., Gao F.Q., Shen X.H., Zhang Q.R., Zhang X.F., Zhou J.X., Gao F.M. (2013). Self-assembly and nanostructures in organogels based on a bolaform cholesteryl imide compound with conjugated aromatic spacer. Materials.

[40] Jiao T., Huang Q., Zhang Q., Xiao D., Zhou J., Gao F. (2013). Self-assembly of organogels via new luminol imide derivatives: Diverse nanostructures and substituent chain effect. Nanoscale Res. Lett..

[41] Jiao T.F., Wang Y.J., Zhang Q.R., Zhou J.X., Gao F.M. (2013). Regulation of substituent groups on morphologies and self-assembly of organogels based on some azobenzene imide derivatives. Nanoscale Res. Lett..

[42] Jiao T.F., Gao F.Q., Zhang Q.R., Zhou J.X., Gao F.M. (2013). Spacer effect on nanostructures and self-assembly in organogels via some bolaform cholesteryl imide derivatives with different spacers. Nanoscale Res. Lett..

[43] Xing R., Liu K., Jiao T., Zhang N., Ma K., Zhang R., Zou Q., Ma G., Yan X. (2016). An injectable self-assembling collagen-gold hybrid hydrogel for combinatorial antitumor photothermal/photodynamic therapy. Adv. Mater..

[44] Jiao T., Zhao H., Zhou J., Zhang Q., Luo X., Hu J., Peng Q., Yan X. (2015). The self-assembly reduced graphene oxide nanosheet hydrogel fabrication by anchorage of chitosan/silver and its potential efficient application toward dyes degradation for wastewater treatments. ACS Sustain. Chem. Eng..

[45] Zhu X., Duan P., Zhang L., Liu M. (2011). Regulation of the chiral twist and supramolecular chirality in co-assemblies of amphiphilic L-glutamic acid with bipyridines. Chem. Eur. J..

[46] Duan P., Qin L., Zhu X., Liu M. (2011). Hierarchical self-assembly of amphiphilic peptide dendrons: Evolution of diverse chiral nanostructures through hydrogel formation over a wide pH range. Chem. Eur. J..

[47] Nayak M.K. (2011). Functional organogel based on a hydroxyl naphthanilide derivative and aggregation induced enhanced fluorescence emission. J. Photochem. Photobiol. A Chem..

[48] Atsbeha T., Bussotti L., Cicchi S., Foggi P., Ghini G., Lascialfari L., Marcelli A. (2011). Photophysical characterization of low-molecular weight organogels for energy transfer and light harvesting. J. Mol. Struct..

[49] Shimizu T., Masuda M. (1997). Stereochemical effect of even-odd connecting links on supramolecular assemblies made of 1-glucosamide bolaamphiphiles. J. Am. Chem. Soc..

[50] Kogiso M., Ohnishi S., Yase K., Masuda M., Shimizu T. (1998). Dicarboxylic oligopeptide bola-amphiphiles: Proton-triggered self-assembly of microtubes with loose solid surfaces. Langmuir.

[51] Wang T.Y., Li Y.G., Liu M.H. (2009). Gelation and self-assembly of glutamate bolaamphiphiles with hybrid linkers: Effect of the aromatic ring and alkyl linkers. Soft Matter.

[52] Zhao W., Li Y., Sun T., Yan H., Hao A., Xin F., Zhang H., An W., Kong L., Li Y. (2011). Heat-set supramolecular organogels composed of β-cyclodextrin and substituted aniline in *N*,*N*-dimethylformamide. Colloid Surf. A-Physicochem. Eng. Asp..

[53] Li Y.G., Wang T.Y., Liu M.H. (2007). Ultrasound induced formation of organogel from a glutamic dendron. Tetrahedron.

[54] He P., Liu J., Liu K., Ding L., Yan J., Gao D., Fang Y. (2010). Preparation of novel organometallic derivatives of cholesterol and their gel-formation properties. Colloid Surf. A Physicochem. Eng. Asp..

[55] Wu J.C., Yi T., Xia Q., Zou Y., Liu F., Dong J., Shu T.M., Li F.Y., Huang C.H. (2009). Tunable gel formation by both sonication and thermal processing in a cholesterol-based self-assembly system. Chem. Eur. J..

[56] Xing R., Jiao T., Ma K., Ma G., Möhwald H., Yan X. (2016). Regulating cell apoptosis on layer-by-layer assembled multilayers of photosensitizer-coupled polypeptides and gold nanoparticles. Sci. Rep. UK.

[57] Jiao T., Liu M. (2006). Supramolecular assemblies of a new series of gemini-type Schiff base amphiphiles at the air/water interface: In situ coordination, interfacial nanoarchitectures, and spacer effect. Langmuir.

[58] Bhattarai N., Gunn J., Zhang M. (2010). Chitosan-based hydrogels for controlled, localized drug delivery. Adv. Drug Deliv. Rev..

[59] Yang H., Yi T., Zhou Z., Zhou Y., Wu J., Xu M., Li F., Huang C. (2007). Switchable fluorescent organogels and mesomorphic superstructure based on naphthalene derivatives. Langmuir.

